# Supplementary material for: Towards automation of the polyol process for the synthesis of silver nanoparticles
Source: Sci Rep. 2022 Apr 6;12:5769. doi: 10.1038/s41598-022-09774-w (PMC8986771; doi:10.1038/s41598-022-09774-w)
Supplement: Supplementary file 1 — Supplementary Information. [file 41598_2022_9774_MOESM1_ESM.docx]

**Supplementary Information**

**for**

***Towards automation of the polyol process for the synthesis of silver nanoparticles***

*Jakob Wolf^1,2^, Tomasz M. Stawski^1^*, Glen J. Smales^1^, Andreas F. Thünemann^1^,*
*Franziska Emmerling^1,3^***

* [tomasz.stawski@bam.de](mailto:tomasz.stawski@bam.de); ** [franziska.emmerling@bam.de](mailto:**franziska.emmerling@bam.de)

^1^Federal Institute for Materials Research and Testing (BAM), 12489, Berlin, Germany;

^2^Max Planck Institute of Colloids and Interfaces, 14476 Potsdam, Germany;

^3^Humboldt-Universität zu Berlin, Department of Chemistry, 12489, Berlin, Germany

***Supplementary files***

1. The graphml file: **reaction_graph_AgNP.graphml** is deposited at ref.^1^. It contains topological information (Fig. 1 in the main text) about the reaction setup and metadata with reaction condtions. It used by the Python API used to control the Chemputer.
2. SAXS reports. The complete report sheets generated by McSAS are deposited at ref.^1^ . They contain extended information characterising the size distributions and the fitting parameters.
   NP3_I: **saxs_report_NP3_I.pdf**

NP3_II: **saxs_report_NP3_II.pdf**

NP3_III: **saxs_report_NP3_III.pdf**

NP3_IV: **saxs_report_NP3_IV.pdf**

NP5_I: **saxs_report_NP5_I.pdf**

NP5_II: **saxs_report_NP5_II.pdf**

NP5_III: **saxs_report_NP5_III.pdf**

***Supplementary figures:***


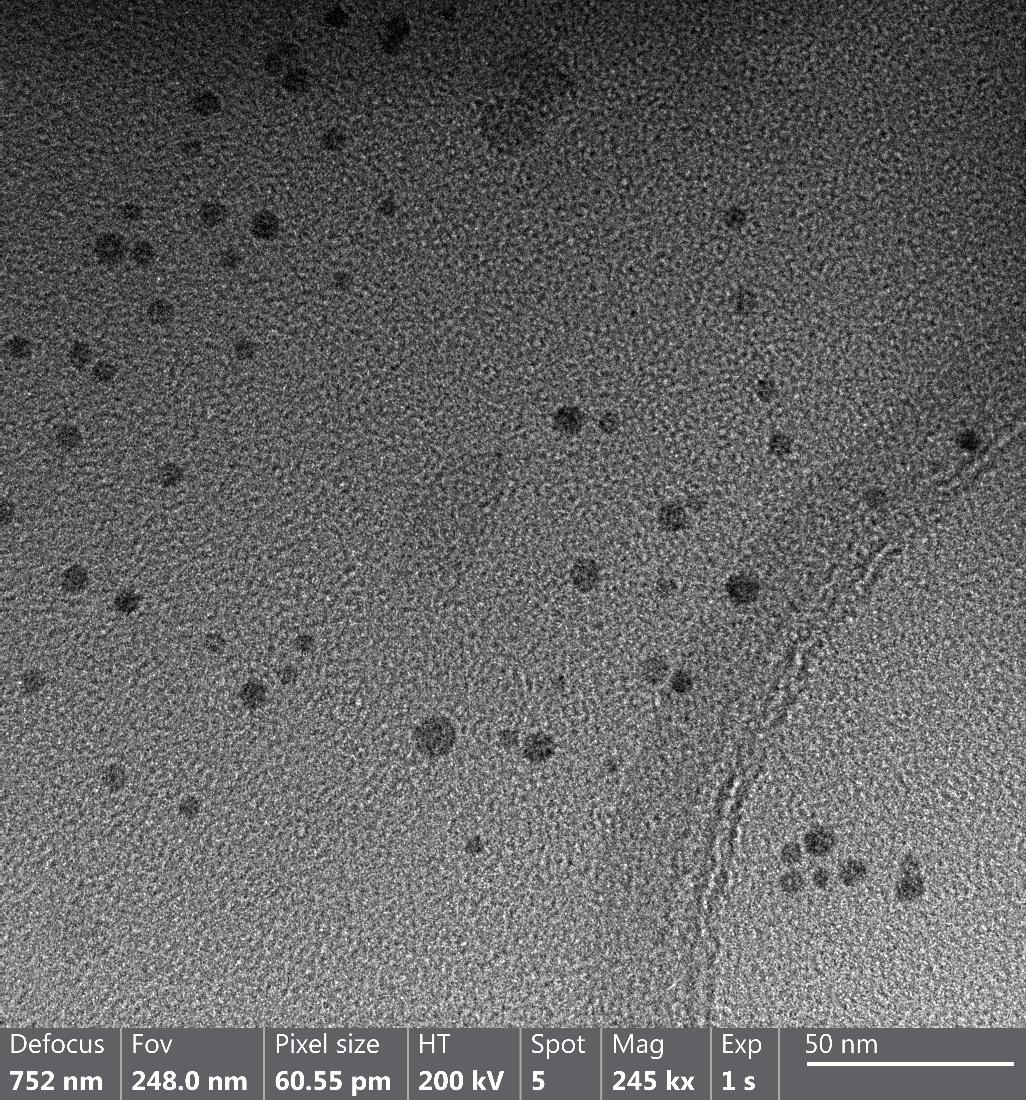


Fig. S1. Bright-field TEM image of the NP3 series nanoparticles. It shows that the NPs were indeed spherical and relatively monodisperse.

Fig. S2. Volume-weighted size distributions for the NP3 (upper) and NP5 (lower) series derived from the SAXS measurements. The mean and standard deviation values are reported in Table 2 in the main text.

Fig. S3. Intensity-weighted size distributions for the NP3 (upper) and NP5 (lower) series derived from the DLS measurements.

Fig. S4. XRD from dried NP3_I (upper) and NP5_I (lower) samples. The black curves represent measured data and the red ones fitted profiles. Both diffraction patterns unequivocally correspond to single-phase Ag (cubic, F m -3 m). Miller indices of the diffraction peaks are provided.

***Supplementary computer code***

**Snippet 1.** Cleaning and priming of the Chemputer.

chempiler.move('AgNO3 in glycol', 'waste reaction', priming_volume, initial_pump_speed=default_speed_viscous)
# priming_volume = 1.5 mL, default_speed_viscous = 5 mL/min
chempiler.move('polyacrylic acid in glycol', 'waste reaction', priming_volume, initial_pump_speed=default_speed_viscous)

# make sure glycol is washed away for chemical compatibility
for i in range(3):
 chempiler.move('water', 'waste reaction', default_cleaning_volume)
# default_cleaning_volume = 5 mL

for i in range(3):
 chempiler.move('HNO3 (aq)', 'cleaning pump', default_cleaning_volume)
 chempiler.move('cleaning pump', 'waste HNO3', default_cleaning_volume)

for i in range(3):
 chempiler.move('water', 'cleaning pump', default_cleaning_volume)
 chempiler.move('cleaning pump', 'waste HNO3', default_cleaning_volume)

chempiler.move('water', 'waste reaction', default_cleaning_volume,repeats=3)
chempiler.move('acetone', 'waste reaction', default_cleaning_volume, repeats=3)
chempiler.move('air', 'waste reaction', default_cleaning_volume, repeats=3)

**Snippet 2.** Synthesis initialisation.

chempiler.move('polyacrylic acid in glycol', 'reactor', 12.5, initial_pump_speed=default_speed_viscous)

# ensure quantitative transfer
chempiler.move('air', 'reactor', 5)

# clean up
chempiler.move('water', 'waste reaction', 5, repeats=3)
chempiler.move('acetone', 'waste reaction', 5, repeats=3)
chempiler.move('air', 'waste reaction', 5, repeats=3)

# heat reactor
chempiler.stirrer.set_temp('hotplate', 210)
chempiler.stirrer.set_stir_rate('hotplate', default_stirring_rate)

# default stirring rate = 400 rpm
chempiler.stirrer.stir('hotplate')
chempiler.stirrer.heat('hotplate')

# make sure tubes are dry
chempiler.move('air', 'reactor', 10)
chempiler.move('air', 'waste reaction', 40)

# wait for reaching the reduction temperature
chempiler.stirrer.wait_for_temp('hotplate')

**Snippet 3.** Solution injection.

chempiler.move('AgNO3 in glycol', 'reactor pump', 2.5, initial_pump_speed=default_speed_viscous)
chempiler.move('air', 'reactor pump', 7)
chempiler.move('reactor pump', 'reactor', 9.5, end_pump_speed=50) #this has to happen rapidly

**Snippet 4.** Stopping the reaction.

# wait for reduction
chempiler.wait(reduction_time)

# wait for cooldown
chempiler.stirrer.set_temp('hotplate', room_temperature)

# room_temperature = 25 °C
chempiler.stirrer.wait_for_temp('hotplate')

**Snippet 5.** Decantation procedure.

# automated decanting
for i in range(3):
 chempiler.stirrer.stir('hotplate')
 chempiler.move('water', 'reactor', 34)
 chempiler.stirrer.stop_stir('hotplate')
 chempiler.wait(24*one_hour)
 chempiler.move('reactor', 'waste reaction', 35)

**Snippet 6.** Final steps.

# automated dilution
chempiler.stirrer.stir('hotplate')
chempiler.move('water', 'reactor', 6.4)

***Supplementary references***

1. Wolf, J., Stawski, T. M., Smales, G. J., Thünemann, A. F. & Emmerling, F. SI Files for ‘Towards automation of the polyol process for the synthesis of silver nanoparticles’. (2022) doi:10.5281/zenodo.5910614.
